# The identification of treatment-resistant depression patients in electronic health records, a retrospective cohort study in China

**DOI:** 10.1192/j.eurpsy.2022.687

**Published:** 2022-09-01

**Authors:** S. Dong, T. Wu, W. Dong, T. Si

**Affiliations:** 1 Janssen Research and Development, Epidemiology, Shanghai, China; 2 Janssen Research and Development, Epidemiology, Beijing, China; 3 Peking University Sixth Hospital, Institute Of Mental Health, Beijing, China

**Keywords:** Treatment-resistant depression, Elctronic Health Records (EHR), Epidemiology, psychiatry

## Abstract

**Introduction:**

Previous Electronic Health Records (EHR) based studies adopted various definitions in identifying Treatment-Resistant Depression (TRD) patients. There is a lack of similar attempts among Chinese population which limits the understanding of TRD in China.

**Objectives:**

Assess TRD identification using EHR from a major psychiatric hospital in China.

**Methods:**

This study utilized a retrospective Major Depressive Disorder (MDD) cohort of patients who newly initiated pharmaceutical treatment (2010-2018); follow-up was ended upon 1-year or treatment discontinuation (≥120d without treatment). TRD was first identified based on common clinical definition of two prior regimen failures (change of regimen) with 4-week as regimen adequacy threshold (Def1). Alternative adequacy thresholds of 2-week and 6-week were applied. Based on Def1 (4-week), at least 3 distinctive regimens were additionally required in TRD identification (Def2). Further, a data-driven definition (Def3) based on drug count as having ≥3 antidepressants or ≥1 antipsychotic within 1 year was considered (Cepeda et al., 2018).

**Results:**

From 12257 MDD patients included in the cohort, Def1 identified 633 (5.2%) TRD cases, whereas regimen adequacy thresholds of 2-week and 6-week identified 1772 (14.5%) and 61 (0.5%) cases, respectively. Further, Def2 identified 261 (2.4%) TRD cases. Finally, Def3 yielded 2449 (20.0%) TRD cases, including 1966 exclusive cases that were not identified by Def1.

**Conclusions:**

This study showed different definitions for TRD identification had considerable impact on the number of patients identified among Chinese population, obscuring the comparability among EHR-based TRD studies. As first step, we found the criteria of regimen adequacy as major contributor to the observed variability in China.

**Disclosure:**

No significant relationships.

